# A how-to guide for a precision medicine approach to the diagnosis and treatment of Alzheimer's disease

**DOI:** 10.3389/fnagi.2023.1213968

**Published:** 2023-08-17

**Authors:** Gayatri Devi

**Affiliations:** ^1^Neurology and Psychiatry, Zucker School of Medicine, Hempstead, NY, United States; ^2^Neurology and Psychiatry, Lenox Hill Hospital, New York City, NY, United States; ^3^Park Avenue Neurology, New York City, NY, United States

**Keywords:** Alzheimer's disease, treatment and diagnosis, precision medicine, differential diagnosis, biomarkers, lecanemab, aducanumab, prognosis

## Abstract

**Article purpose:**

The clinical approach to Alzheimer's disease (AD) is challenging, particularly in high-functioning individuals. Accurate diagnosis is crucial, especially given the significant side effects, including brain hemorrhage, of newer monoclonal antibodies approved for treating earlier stages of Alzheimer's. Although early treatment is more effective, early diagnosis is also more difficult. Several clinical mimickers of AD exist either separately, or in conjunction with AD pathology, adding to the diagnostic complexity. To illustrate the clinical decision-making process, this study includes de-identified cases and reviews of the underlying etiology and pathology of Alzheimer's and available therapies to exemplify diagnostic and treatment subtleties.

**Problem:**

The clinical presentation of Alzheimer's is complex and varied. Multiple other primary brain pathologies present with clinical phenotypes that can be difficult to distinguish from AD. Furthermore, Alzheimer's rarely exists in isolation, as almost all patients also show evidence of other primary brain pathologies, including Lewy body disease and argyrophilic grain disease. The phenotype and progression of AD can vary based on the brain regions affected by pathology, the coexistence and severity of other brain pathologies, the presence and severity of systemic comorbidities such as cardiac disease, the common co-occurrence with psychiatric diagnoses, and genetic risk factors. Additionally, symptoms and progression are influenced by an individual's brain reserve and cognitive reserve, as well as the timing of the diagnosis, which depends on the demographics of both the patient and the diagnosing physician, as well as the availability of biomarkers.

**Methods:**

The optimal clinical and biomarker strategy for accurately diagnosing AD, common neuropathologic co-morbidities and mimickers, and available medication and non-medication-based treatments are discussed. Real-life examples of cognitive loss illustrate the diagnostic and treatment decision-making process as well as illustrative treatment responses.

**Implications:**

AD is best considered a syndromic disorder, influenced by a multitude of patient and environmental characteristics. Additionally, AD existing alone is a unicorn, as there are nearly always coexisting other brain pathologies. Accurate diagnosis with biomarkers is essential. Treatment response is affected by the variables involved, and the effective treatment of Alzheimer's disease, as well as its prevention, requires an individualized, precision medicine strategy.

## Introduction

Alzheimer's disease (AD) is a complex and heterogenous disorder characterized by synaptic dysfunction in crucial brain networks, accompanied by the presence of intracellular neurofibrillary tangles and extracellular amyloid plaques, as well as glial inflammatory changes (Deture and Dickson, 2019). While the precise mechanisms by which these pathological events cause the clinical symptoms of AD are not entirely understood, the normal brain's continuous synaptic pruning by microglia goes awry (Knopman et al., 2021).

AD is best conceptualized not as a single monolithic disease but as a syndromic disorder, with varying neurocognitive profiles and prognoses based on the brain regions affected (Devi, 2018a). While memory impairment, with associated hippocampal involvement, is the most frequent complaint, impairment of other cognitive domains, including language, maybe the presenting complaint. Based on the subtype of Alzheimer's, progression can vary from a decline of 1 point per year on the mini-mental state examination to 5 points annually (Murray et al., 2011).

**Figure 1:**
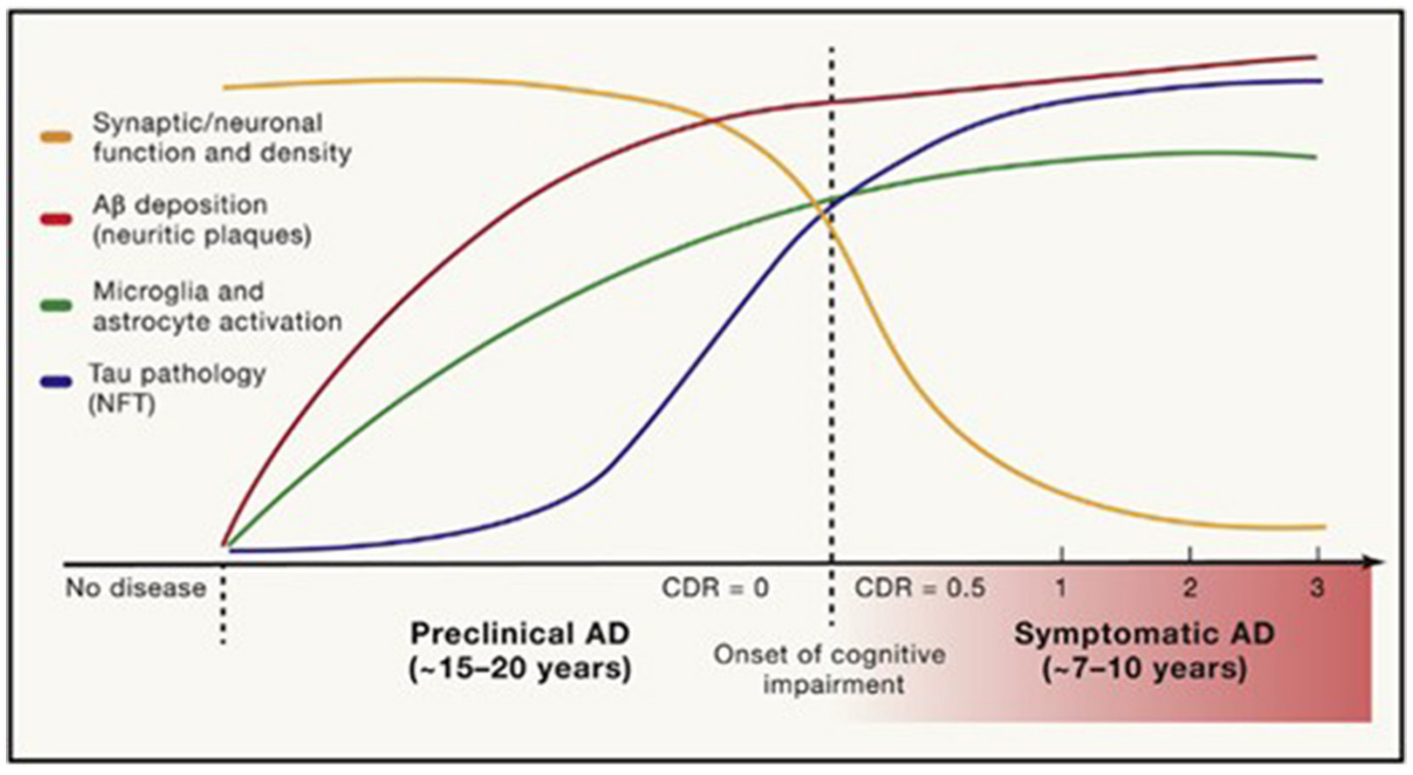
Timing of pathological and clinical events in AD. Long and Holtzman (2019) in Cell 179, used with author-permitted text adaptation and publisher permission.

The prodromal stage of AD, with minimal-to-mild cognitive impairment without significant functional dysfunction, may last for years (Figure 1) (Long and Holtzman, 2019). The progressive loss of synapses and subsequent failure of eloquent cortical networks occur over decades, providing a long window of opportunity for the prevention of clinical disease and intervention with disease-modifying therapies. The age of onset, genetic risk, co-existing primary brain pathologies (such as cerebrovascular disease, argyrophilic grain disease, or Lewy body disease), systemic comorbidities (including cardiovascular disease), psychiatric co-pathology, an individual's brain reserve and cognitive reserve, as well as educational and socioeconomic factors, all influence the symptoms, progression, and treatment response in AD.

Diagnosing AD based solely on clinical symptoms lacks sensitivity and specificity due to the overlap of symptoms with normal aging and the routine occurrence of concomitant primary brain pathology with similar clinical presentations, as AD occurring in isolation is a rarity. Multiple other primary brain conditions routinely co-exist with AD (Hampel et al., 2018). Biomarkers play a crucial role in accurately diagnosing AD and guiding appropriate care. Even in specialized memory disorder centers, before the advent of biomarkers, the positive predictive value on autopsy, of a clinical diagnosis of AD, ranged from 46% to 83% and one-third of patients in clinical trials did not have AD pathology (Beach et al., 2012). The reverse is also true, with patients being misdiagnosed with other conditions when, in fact, they have AD. As many as a quarter of patients with a clinical diagnosis of frontotemporal dementia, for instance, turn out to have AD instead on biomarker analysis, with important therapeutic and prognostic implications (Padovani et al., 2013).

Currently available treatments, when used in a biologically complementary manner tailored to the individual patient, may be more effective in helping slow disease progression. This may be especially the case in older-age AD, which is typically multifactorial in etiology and comprises 95% of cases (Devi and Scheltens, 2018). Treatment success may be pragmatically defined as maintaining functional independence until death, even if the primary pathology remains unchanged.

An n-of-1, precision medicine approach to the prevention and treatment of AD, taking into consideration the unique characteristics of each patient, can optimize outcomes and improve the potential for maintaining independent functioning (Arafah et al., 2023). To illustrate this approach to treating AD in a clinical setting, actual patients are presented, highlighting both diagnostic and treatment dilemmas, as well as illustrative therapeutic responses.

## Prevalence and risk factors

Estimating the true prevalence of AD is problematic due to numerous factors, including the evolving definition of the disease, different methods used for diagnosis (clinical vs. biomarker-based), presence of confounding variables such as co-pathologies, patient characteristics such as education and socioeconomic status, and diagnosing physician characteristics such as specialty. However, a widely accepted estimate places prevalence at ~1 in 9 people at and above the age of 65 years, with the proportion increasing with age.

Age is the greatest risk factor for sporadic, older-age Alzheimer's disease. Gender is another risk factor, as more women than men have AD. Men succumb earlier to cardiovascular disease, which may be one explanation for the higher prevalence of AD in women (Podcasy and Epperson, 2016). The lack of estrogen in post-menopausal women may also increase the risk for AD which may be ameliorated by estrogen use (Henderson, 2006; Saleh et al., 2023).

Genetics plays a role, with the ε4 isoform of the apolipoprotein E (APOE) gene being an established risk factor. A single copy of the ε4 allele increases the risk for older-age AD by three- to four-fold when compared with ε3 carriers, while two copies increased the risk 12–15-fold (Knopman et al., 2021). APOE genotyping has become clinically more relevant with the advent of newer therapies, as patients with an ε4 allele are at higher risk of serious side effects (Salloway et al., 2022; Devi, 2023). Rare protein-damaging variants in other genes, such as *SORL1, ABCA7*, and *TREM2*, have also been associated with an increased risk for AD (Scheltens et al., 2021). Mutations in the presenilin (PSEN) 1 gene on chromosome 14 and the PSEN2 gene on chromosome 1 are the most common *cause*, not a *risk*, for younger-age AD. The terms younger- and older-age AD arbitrarily divided at age 60 or 65 years, are used here in lieu of early-onset and late-onset AD, as patients and families often conflate the latter terms with earlier and later disease stages.

Modifiable risk factors, which can be addressed through lifestyle changes, may further increase the risk for older-age AD (Knopman et al., 2021). Cardiovascular disease, cerebrovascular disease, diabetes mellitus, hypertension, obesity, and hyperlipidemia increase the risk for AD. Other modifiable risk factors include hearing and vision loss, traumatic brain injury, smoking, alcohol abuse, depression, reduced physical activity, and reduced social engagement causing isolation and loneliness. Depression and anxiety may confer additional risk for developing AD or may be part of the prodromal phase.

Because AD pathology begins in patients as early as their thirties and forties, but clinical manifestations are delayed until a much older age, intervention in young adulthood would be the preferred strategy to reduce the risk for AD.

## Definition of Alzheimer's disease

The evolving definition of Alzheimer's disease has led to improvements in antemortem diagnostic accuracy, particularly with the availability of neuroimaging and laboratory biomarkers. Historically, criteria for diagnosing Alzheimer's disease focused on excluding other causes of dementia, such as stroke (McKhann et al., 1984). The European International Working Group (IWG) proposed incorporating biomarkers into the diagnostic framework (Dubois et al., 2010). Further refinements led to the two current definitions for AD—the American radiologist-led purely biomarker-based amyloid, tau, and neurodegeneration (ATN) on neuroimaging definition proposed in 2018 and the 2021 European neurologist-led IWG definition that requires functional impairment in addition to biomarkers (Jack et al., 2018; Dubois et al., 2021).

There is debate about the sole use of biomarkers to diagnose AD. While the ATN premise is that individuals with positive biomarkers will surely develop clinical symptoms given enough time and may benefit from early enrollment in clinical trials, the IWG perspective is that the presence of biomarkers does not necessarily equate to Alzheimer's, as biomarker-positive persons may remain asymptomatic. To avoid giving a potentially devasting diagnosis to an asymptomatic person, the IWG proposes classifying asymptomatic individuals with positive biomarkers as “asymptomatic, with features associated with future development of AD,” rather than diagnosing them with Alzheimer's disease (Dubois et al., 2021).

Amyloid plaques, one of the biomarkers used to diagnose AD, are present in 20% of cognitively normal persons in their 60 s, one-third of those 70 years and over, and half of those 95 years and older (Jansen et al., 2022). Additionally, <5% of 76-year-olds with cognitive complaints and amyloid plaques progress to AD after 2.5 years (Dubois et al., 2018). Such data support a combined clinical-biomarker approach, as proposed by the IWG, avoiding over diagnosis, and yet identifying candidates for intervention.

## Neuropathology in Alzheimer's disease (tau, aβ protein, and neuroinflammation)

The neuropathological hallmarks of Alzheimer's disease are extracellular plaques composed of Aβ protein and intracellular tangles composed of abnormally phosphorylated tau (Knopman et al., 2021). First described by the German psychiatrist Alois Alzheimer in 1906, his department chairman Emil Kraepelin named the condition after him (Devi and Quitschke, 1999). Inflammatory glial changes are now further considered part of AD pathology.

**Figure 2:**
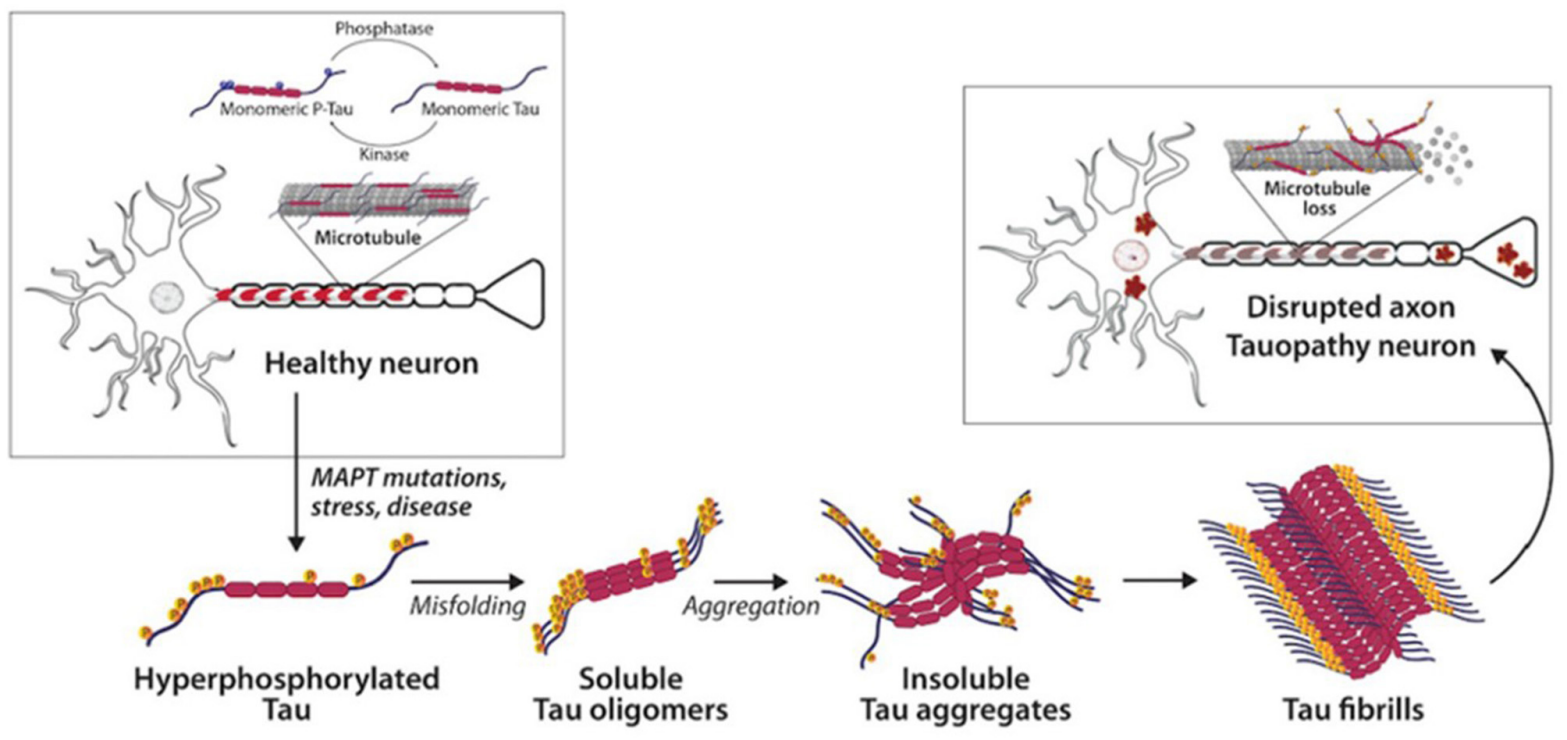
Tau and neurofibrillary tangles in health and disease (Catarina Silva and Haggarty, 2020).

**Figure 3:**
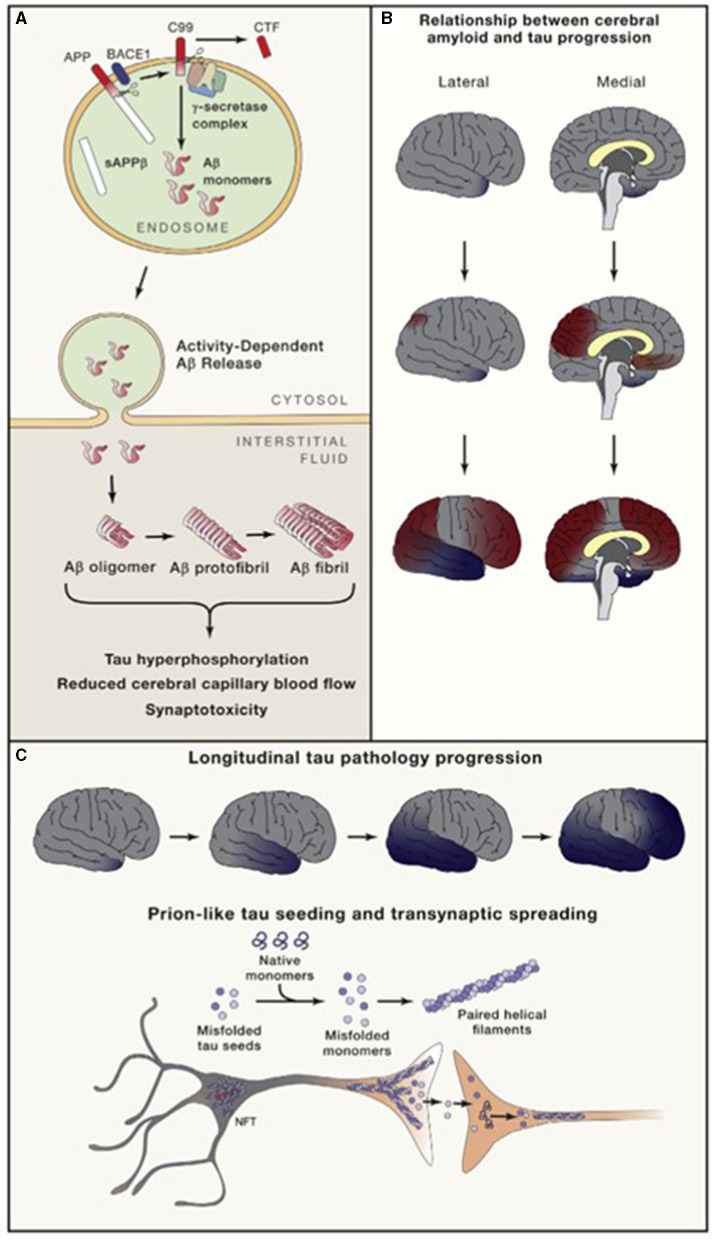
Amyloid **(A)** and neurofibrillary tangle production and direction of cortical spread of pathology **(B, C)**. Long and Holtzman (2019) in Cell 179 used with author-permitted text adaptation and publisher permission. APP, Amyloid precursor protein; BACE, Beta amyloid cleaving enzyme; CTF, C-terminal fragment; NFT, Neurofibrillary tangles; sAPP, soluble APP.

Both tau and Aβ proteins are highly disordered and unstructured, and therefore prone to instability and pathologic misfolding, which processes are exacerbated by aging (Hipp et al., 2019). These pathologic protein confirmations behave like seeds, instigating further pathologic misfolding of normal tau and Aβ proteins, with the changes propagating across cells and spreading pathology, as seen in prion diseases (Plotkin and Cashman, 2020). Tau normally maintains cell structure, axonal transport, and synaptic function. Abnormal hyperphosphorylation of tau disrupts these functions by forming insoluble, intracellular tangles, leading to neurotoxicity (Figure 2). Similarly, Aβ protein of varying lengths is derived from the larger amyloid precursor protein (APP) and is involved in neuroprotection and synaptic function. However, cleavage of APP by γ and β secretase enzymes produces longer, primarily Aβ-42 amino acid chains which assemble into oligomers, then protofibrils and amyloid fibrils, which ultimately assemble into amyloid plaques (Figure 3).

Although amyloid plaque density correlates with AD severity, it is generally accepted that the plaques are likely neuroprotective, sequestering and neutralizing the component, longer-chain, rogue Aβ oligomers which are neurotoxic (Tamagno et al., 2018). That Aβ oligomers, rather than plaques, drive AD pathology is supported by the “Osaka” mutation, where patients have a rapid onset of AD with characteristic Aβ and tau levels in CSF without the presence of plaques (Tomiyama and Shimada, 2020). Intriguingly, tau aggregation into neurofibrillary tangles may also be protective with soluble, oligomeric tau being neurotoxic (Morsch et al., 1999; Takeda et al., 2016).

A critical role of Aβ in the pathogenesis of AD is clear from the fact that mutations in genes related to Aβ production and clearance are involved in *all* genetic causes of AD such as trisomy 21, presenilin (PSEN)1, and PSEN2 mutations. Additionally, the ε4 isoform of the APOE gene, associated with a greater risk for older-age AD, is less efficient at clearing Aβ than the ε2 and ε3 isoforms (Deture and Dickson, 2019). Interestingly, although abnormal tau is clearly implicated in AD pathogenesis, mutations in the gene instructing tau production, the MAPT gene, are not associated with Alzheimer's disease.

Tau and amyloid pathology are present in a benign fashion in various brain areas and are deposited in a chronologically asynchronous fashion. However, when a certain amyloid threshold burden is reached, which varies among persons, tau begins to spread pathologically, also in varying patterns, ultimately leading to each person's n-of-1 specific Alzheimer's disease state (Karran and De Strooper, 2022).

As tau accumulates closer to the time of symptom onset and continues to accumulate as the disease progresses, while amyloid plaques begin accumulating decades before symptoms, plaque deposition may be viewed as more disease-specific, and tau deposition as more stage-specific (Knopman et al., 2021) (Figure 3). There may also be pathologic interaction between the two, causing further aggregation.

Additionally, neuro-inflammation plays a critical role in AD pathogenesis, contributing to neurodegeneration and disease progression. Alterations in microglia and astroglia lead to neuro-inflammation, alterations in vasculature, and dysfunction of the glymphatic system, acting in tandem with or before Aβ accumulation, as a driver and component of AD neuropathology (Deture and Dickson, 2019).

## Differential diagnosis; other primary brain pathologies coexisting with Alzheimer's disease

Several other primary brain pathologies commonly coexist with AD, making diagnosis, prognosis, and response to treatment more complex. This is important from a diagnostic, therapeutic, and prognostic perspective, as patients with AD or another primary brain disease such as cerebrovascular disease or normal pressure hydrocephalus nearly always have a concomitant diagnosis that needs monitoring and assessment for treatment.

It is easy to see why, with increasing age, there is a higher co-prevalence of common aging-related brain diseases such as vascular dementia. However, younger-age AD also has a high association with other primary brain disease. This suggests that the neurodegenerative pathology that drives AD may be a catalyst for other primary brain pathologies, regardless of age.

One study found that 98% of younger-age AD and 100% of older-age AD had additional primary brain pathologies, including argyrophilic grain disease and vascular disease (Spina et al., 2021). As compared to younger-age AD, older-age AD patients were more likely to have common co-pathologies, including limbic-predominant, age-associated transactive response DNA-binding protein of 43 kDa (TDP-43) encephalopathy (8% vs. 35%), hippocampal sclerosis (3% vs. 15%), argyrophilic grain disease (41% vs. 58%), and vascular brain injury (39% vs. 65%) (Spina et al., 2021).

In an autopsy series of neuropathological AD, only one-third had AD-only pathology (and even within this group, between 30% and 50% had at least one infarct), 50% had additional TDP-43 pathology, 22% had additional α-synuclein pathology, and 18% had additional combined α-synuclein and TDP-43 pathology (Karanth et al., 2020).

While Alzheimer's, cerebrovascular disease, Parkinson's, and normal pressure hydrocephalus have biomarker-based laboratory or neuroimaging antemortem diagnosis available, other neurodegenerative primary brain pathologies do not as yet.

### Cerebrovascular disease

There is nearly always some level of cerebrovascular disease (CVD) in patients with older-age AD, as both conditions become increasingly more common with age. Up to 84% of persons who die in their 80s have some level of CVD, most often cerebral amyloid angiopathy and small vessel disease. Cerebral amyloid angiopathy results from Aβ deposition in brain parenchymal arteries, arterioles, and capillaries. Vascular pathology may directly increase Aβ deposition by inducing accumulation and impeding clearance (Attems and Jellinger, 2014). Both CVD and AD share many risk factors aside from age, including hypertension, diabetes mellitus, smoking, hypercholesterolemia, and cardiovascular issues such as atrial fibrillation. Such sharing of risk factors in both conditions led to a provocative, highly cited article suggesting that Alzheimer's disease be considered a cerebrovascular disorder (De La Torre, 2002).

### Normal pressure hydrocephalus

In a large autopsy series of persons with a clinical diagnosis of normal pressure hydrocephalus (NPH), 74% had evidence of other primary brain neuropathology, with AD contributing to 53% of these cases (Cabral et al., 2011). While NPH is present in 5–9% of those in their 80s and older, the classic triad of cognitive disturbance, a broad-based shuffling gait, and urinary incontinence is present in <60% of patients (Jaraj et al., 2014; Graff-Radford and Jones, 2019; Müller-Schmitz et al., 2020). Additionally, multiple factors contribute to gait disturbance in the elderly, such as lower extremity arthritis and neuropathy (Graff-Radford and Jones, 2019). Neuroimaging revealing enlarged subarachnoid space and a tight high convexity in addition to ventriculomegaly, which is common in the elderly, is supportive of NPH (Graff-Radford and Jones, 2019). Even those patients who improved initially with shunting over the first few years deteriorate with time, suggesting a shared basis for neurodegenerative NPH and AD (Müller-Schmitz et al., 2020).

### TDP-43 pathology and hippocampal sclerosis

Transactive response DNA binding protein of 43 kDa (TDP-43) regulates gene expression, and truncated forms of TDP-43 are found in several neurodegenerative diseases. Limbic predominant, age-associated, TDP-43 encephalopathy (LATE) is as prevalent as AD in persons 80 years and older. LATE presents as a dementia syndrome with memory impairment that is clinically indistinguishable from AD. To make matters even more challenging, the diseases often present together, and the prevalence of TDP-43 pathology increases with the severity of AD, presenting in 20–57% during the earlier stages of AD and in 75% of those with more advanced AD (Jo et al., 2020; Meneses et al., 2021).

**Figure 4:**
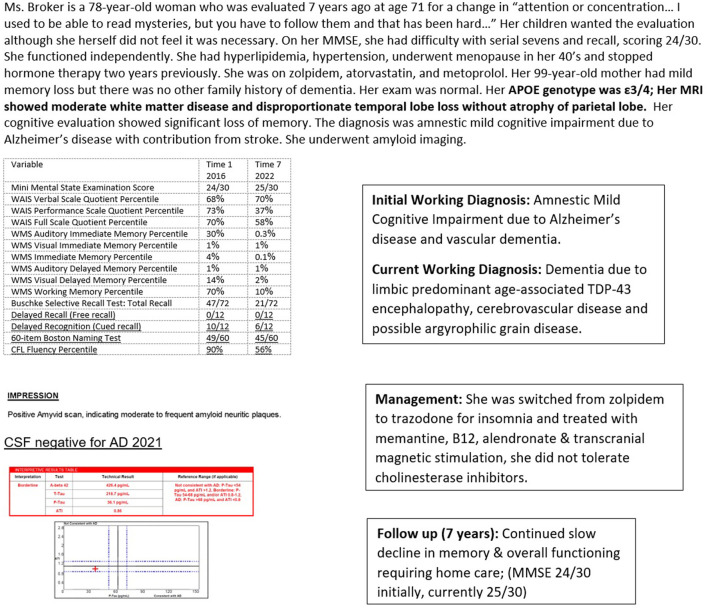
Case 1—Amyloid positive, tau negative, likely limbic predominant age-associated TDP-43 encephalopathy. WAIS, Wechsler Adult Intelligence Scale; WMS, Wechsler Memory Scale.

Case 1 (Figure 4) illustrates likely TDP-43-related LATE pathology in a woman seen at age 71 years and followed over 7 years. She had significant temporal lobe atrophy that spared her parietal cortex along with moderate white matter disease on MRI. She was strongly positive on amyloid imaging with severe memory deficits, scoring at the lowest 1st percentile on nearly all memory domains. She was initially treated as having mild cognitive impairment due to Alzheimer's pathology and vascular disease. When aducanumab became commercially available, a spinal tap was done to confirm the diagnosis of AD. The spinal fluid analysis revealed a low cerebrospinal fluid Aβ42, consistent with AD and consistent with brain amyloid deposition seen on her earlier neuroimaging. However, her low level of cerebrospinal p-tau was inconsistent with a diagnosis of AD. She has remained stable over several years without deterioration in language and visuospatial or physical skills, despite even further diminution of memory. Her mini-mental state examination score has remained stable at 25/30, with an initial score of 24/30 7 years previously.

In persons 80 years and older, 20–50% have enough TDP-43 accumulation to cause cognitive loss in a large community-based autopsy series (Jo et al., 2020). Hippocampal sclerosis pathology may be observed but is neither necessary nor sufficient for a diagnosis of LATE.

Neuronal loss and gliosis of the hippocampal formation that is out of proportion to AD-type pathology is termed hippocampal sclerosis of aging (HS-Aging) and is strongly associated with TDP-43 pathology. In one study, 90% of aged persons with HS-Aging pathology exhibited TDP-43 pathology while only 10% of controls without HS-Aging did (Nelson et al., 2019). HS-Aging is present in 5–30% of nonagenarians along with astrocytic changes that are a histopathologic feature of HS-Aging. Hippocampal sclerosis due to epilepsy or vascular insufficiency does not stain for TDP-43.

### α-synuclein pathology: lewy body disease and Parkinson's disease

Lewy bodies are abnormal intracellular inclusions of α-synuclein, a protein that normally regulates neurotransmitter release. Based on the brain regions affected, Lewy bodies lead to Parkinson's disease (PD), Parkinson's disease dementia (PDD), and dementia due to Lewy bodies (DLB). The distinction between PDD and DLB is arbitrarily based on the difference in time of onset between motor and cognitive symptoms and not on distinctive neuropathology (Jellinger and Korczyn, 2018). If motor symptoms antedate cognitive symptoms by <1 year, the diagnosis is DLB, and if by more than a year before cognitive symptoms begin, the diagnosis is PDD.

As there are no neuropathological criteria that distinguish DLB from PDD and as the two conditions share genetic risk factors and prodromal features, they may be regarded as a single disease across a spectrum (Walker et al., 2015; Coughlin et al., 2020). Lewy body pathology is found in nearly half of patients with both younger- and older-age AD (Chung et al., 2015; Ferman et al., 2018; Spina et al., 2021).

Patients with DLB present with a history of restless legs, have early visual hallucinations, and depression. Interestingly, they often have insight into their hallucinations (“they seem real at the time, but I know they are not”). Patients have more prominent visuo-spatial disturbances on formal cognitive testing. Resting tremors are rare in patients with DLB and common in patients with PD. Dopamine uptake scans may help distinguish between the two conditions, as one study found that patients with PD show markedly reduced putaminal and asymmetric caudate uptake, while DLB shows nearly absent caudate and putaminal uptake (Nichols et al., 2018). All patients with α-synuclein pathology are more susceptible to the extrapyramidal side effects of dopamine antagonists such as those commonly used to treat hallucinations.

**Figure 5:**
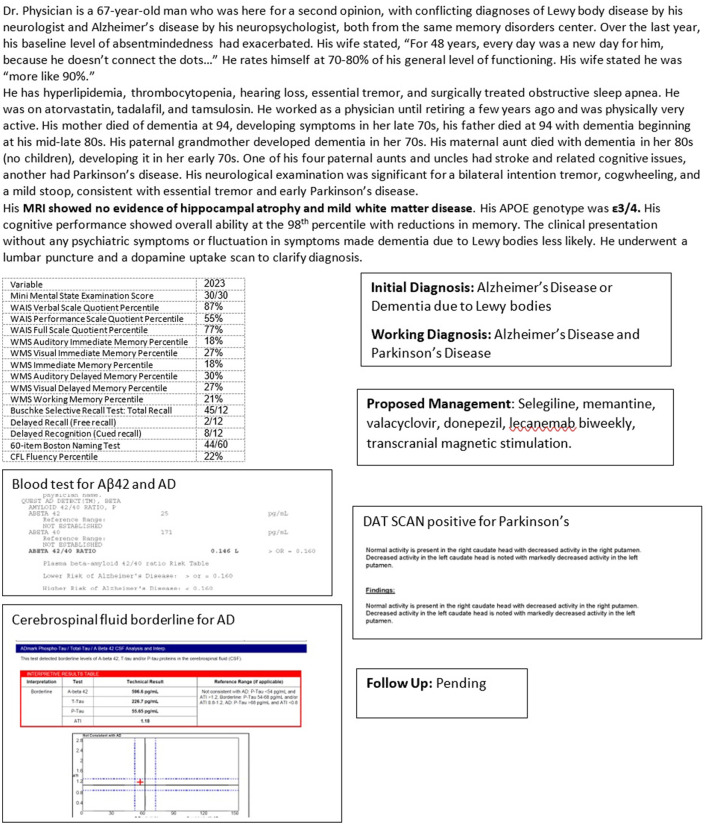
Case 2—Co-pathology in Alzheimer's disease: Lewy body disease, versus Parkinson's disease dementia.

Case 2 (Figure 5) highlights the diagnostic and treatment dilemma in a high-functioning patient, initially diagnosed with Alzheimer's by a neuropsychologist, then as essentially normal or possibly dementia due to Lewy bodies by his cognitive neurologist, now with a diagnosis of AD and PD, based on biomarkers. Such shifts in diagnoses will have obvious treatment consequences.

### Argyrophilic grain disease

Argyrophilic grain disease (AGD) is a widely prevalent primary brain pathology that is often underrecognized. It affects 9% of 65-year-olds, rising to 31% in centenarians (Ferrer et al., 2008). Unless appropriate stains are used, argyrophilic grains may be missed, but with appropriate staining, AGD pathology is seen in over a quarter of all AD cases (Yokota et al., 2018). Most cases of AGD are asymptomatic, although some present with a dementia that is indistinguishable from AD. Case 1 (Figure 4) may have some components of AGD. Personality changes and psychiatric symptoms may be presenting features and AGD should be considered in the differential of late-life psychosis. AGD progresses from the anterior entorhinal cortex to the neocortex and brain stem, as is characteristic of tau propagation (Saito et al., 2004).

### Aging-related tau astrogliopathy and primary age-related tauopathy

Aging-related tau astrogliopathy (ARTAG) may present clinically with focal symptoms such as aphasia when the pathology is regionally limited. A common co-pathology, ARTAG, is found in about 40% of patients with AD post-mortem (Liu et al., 2016). ARTAG is generally seen in persons 60 years and older and is rarely an isolated finding. The pathology is found in astroglia and not in neurons (Kovacs, 2020).

Primary age-related tauopathy (PART) was previously considered normal aging or neurofibrillary tangle predominant senile dementia without significant amyloid pathology (Crary et al., 2014). Cognitively normal persons may exhibit pathologically definite PART. Cognitive impairment in PART is more often seen in those over 80 years of age with a family history of cognitive disorders. Even patients with severe PART can be asymptomatic, although some exhibit mild cognitive loss and rarely, dementia. While ARTAG or PART are generally not clinically relevant in the differential diagnosis of AD, they may affect the progression of AD.

### Menopause-related cognitive impairment (MeRCI)

Cognitive impairment is seen in 60% of menopausal women in their 40 and 50 s (Devi et al., 2005). Subjective memory complaints are associated with objective reductions in verbal, episodic, and list-learning memory, verbal fluency, and executive functioning, indistinguishable from Alzheimer's disease (Henderson and Sherwin, 2007). Estrogen is crucial to brain health and multiple mechanisms drive these deficits. Estrogen increases hippocampal synaptic spine density and synaptic formation, boosts cholinergic and serotonergic function, reduces free radical damage in neurons, reduces cortisol, improves mitochondrial function-important for optimizing brain energy utilization, and improves aspects of cardiovascular health (Toran-Allerand et al., 1999; Davis et al., 2015). Menopause-related cognitive impairment (MeRCI) should be considered in the differential diagnosis of cognitive loss in perimenopausal and menopausal women. A menopausal history is necessary in any memory evaluation of women to avoid misdiagnosis (Devi, 2018b; Devere, 2019).

**Figure 6:**
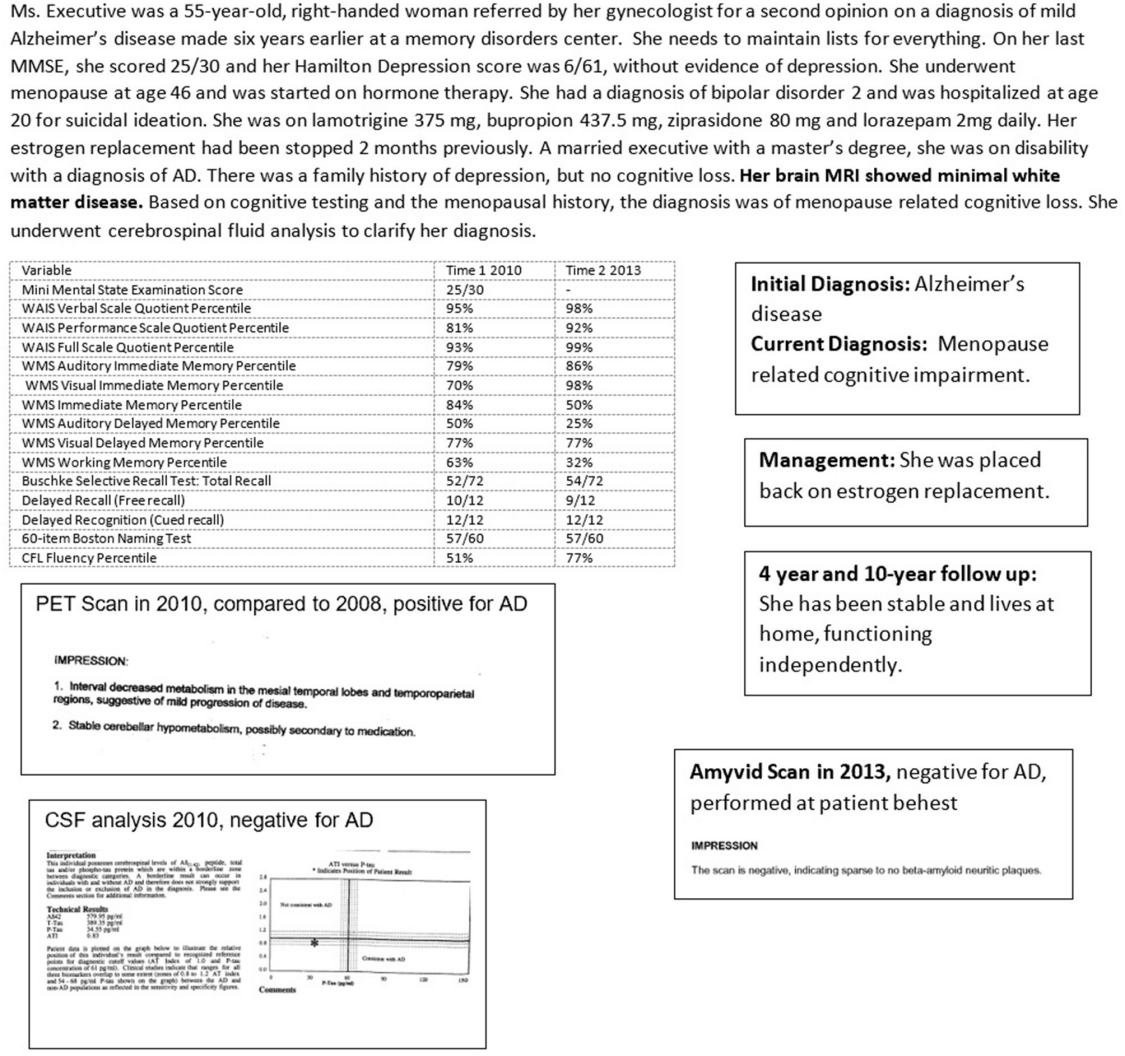
Case 3—An Underrecognized Alzheimer's Disease Mimic: Menopause Related Cognitive Impairment (MeRCI).

Case 3 (Figure 6) concerns a woman diagnosed with AD at an academic memory disorders center, based on cognitive changes and FDG-PET scanning, without taking into consideration her menopausal status (Devi, 2018b). While her neurocognitive performance showed cognitive loss in a pattern that was difficult to distinguish from early AD, as is common in MeRCI, her cerebrospinal fluid biomarkers were negative for AD. Amyloid scanning was performed at her request several years after her cerebrospinal fluid evaluation was negative. At follow-up, nearly a decade later, she remained stable (Devi, 2018b).

### Psychiatric co-morbidities in AD

Early during Alzheimer's disease, anxiety and depression are common psychiatric co-morbidities, with a prevalence of 10–40% (Chemerinski et al., 1998; Botto et al., 2022). Patients often worry about having their deficits discovered, withdraw socially, and stop participating in conversations for fear of being “thought stupid”. Depression is driven by prevailing themes of loss of capacity, institutional placement, and being a familial burden. Conversely, nearly one-third of adults with depression have associated cognitive impairment. Neurochemical changes in cholinergic, serotoninergic, and dopaminergic activity, common to both AD and psychiatric syndromes, drive symptomatology (Botto et al., 2022).

Later in the course of AD, but earlier in the disease if there is concomitant Lewy Body or argyrophilic grain disease co-pathology, there may be paranoia and psychosis. Paranoia is often related to delusions of theft, generally felt to be perpetrated by those physically proximate to the patient, usually the primary caregiver. Concerns about losing financial control are common and understandably exacerbated when the family begins to take control of finances as the patient's capacity declines.

## Accurate diagnosis of Alzheimer's disease

Cognitive dysfunction in AD can be challenging to accurately assess and quantify. Several variables impact the assessment of cognitive function in AD, including patient-specific, physician-specific, and test-specific factors. Patient-specific factors include gender, race, level of education, current level of cognitive demand, brain and cognitive reserve, other primary brain, systemic, and psychiatric co-morbidities, as well as available support systems. Physician-specific variables include specialty, comfort with biomarker usage, expertise with neurocognitive testing, and time available to spend with the patient. Test-specific variables include types of cognitive tests administered, either screening tests or extensive neurocognitive tests, and biomarkers used, whether imaging, blood-based, or cerebrospinal fluid analysis.

The level of routine cognitive demand in a person's daily life, such as being employed vs. retired, can also impact their perception of cognitive dysfunction. A retired person may notice less dysfunction than someone in a new job or someone in a high-cognitive demand position such as law. The availability of ancillary staff or support systems can further impact the ability to maintain functioning (Devi, 2018a).

An approach informed by the patient, physician, and test-specific factors involved is important in obtaining as accurate and reliable an assessment of cognitive function as possible. This is particularly important for early intervention and treatment. Table 1 delineates a comprehensive method for the diagnosis of cognitive disorders.

**Table 1:** Workup of patient of cognitive loss.

**History: Family history of younger and older-age dementia: Unless family members have had autopsy or biomarker-confirmed Alzheimer's disease (AD), it should not be notated as AD**.
Personal history: cardiovascular and cerebrovascular risk factors. History of head injury.
**General medical, neurological, and psychiatric evaluation; Cognitive testing**
Ideally, a comprehensive cognitive battery in persons who score well on screening tests such as the mini-mental state examination or the Montreal cognitive assessment.
**Laboratory workup:**
APOE genotype Younger-age AD mutation screen (for patients with symptoms beginning before age 65 years) Cerebrospinal fluid evaluation for tau and βA-42 analysis β42 amyloid in the blood
**Imaging and other testing:**
Fluorodeoxyglucose positron emission tomography (FDG-PET) may be used to differentiate between frontotemporal dementia and Alzheimer's disease. Positron emission tomography (PET) for amyloid uptake. Computed Tomography (CT) of the brain to evaluate for strokes. Transcranial Doppler may be used to assess blood flow for vascular cognitive impairment. Electroencephalogram may be used to assess change in pattern over time. Magnetic Resonance Imaging (MRI) of the brain evaluates the transependymal fluid flow, stroke, hippocampal atrophy, and general atrophy.

## Neurocognitive evaluation

The type of cognitive assessment administered can affect the determination of cognitive function. Highly educated persons may do well on simple screening tests such as the mini-mental state examination (MMSE) or the Montreal cognitive assessment (MoCA) despite significant cognitive impairment, although the MoCA is more sensitive than the MMSE (Gagnon et al., 2013). On the other hand, highly educated persons may complain of a change in functioning without significant pathology.

Screening tests such as the MMSE or MoCA may also not detect changes in patients with high cognitive reserves or levels of education. Case 2 (Figure 5), a professional, had a perfect MMSE score of 30/30 but could only recall 2/12 items on a more thorough list learning memory task.

A comprehensive neurocognitive assessment that evaluates multiple cognitive domains, including verbal, visual, and executive skills, auditory, visual and working memory, language and verbal fluency, provides a better baseline. However, such testing is expensive and requires skilled personnel to not only administer, but also, separately, interpret the results. An alternative may be to use relatively comprehensive batteries, with ease of use, such as the Cognitive Assessment Toolkit made possible by the Alzheimer's Association (Cordell et al., 2013; Alzheimer's Association, 2018).

## Neuroimaging diagnosis

Neuroimaging techniques used in diagnosing AD and to aid in the differential diagnosis include CT (computed tomography), MRI (magnetic resonance imaging), FDG-PET (fluorodeoxyglucose positron emission tomography), dopamine transporter (DaT) Scan, and amyloid PET scanning. Tau PET scanning is not yet commercially available.

MRI is preferred to CT for better delineation of brain structures, particularly the hippocampus, ability to gauge fluid flow, important for differentiating NPH, and for superior determination of cerebrovascular pathology. Significant hippocampal atrophy may help to diagnose LATE as in Case 1 (Figure 4). FDG-PET scan may reveal characteristic temporoparietal hypometabolism in AD and may also help in differentiating from FTD, although as seen in Case 3 (Figure 6), reliance on FDG-PET as the primary biomarker may lead to misdiagnosis, particularly in atypical presentations. A DaT scan may help to distinguish PD, PDD, and DBLD from AD as illustrated by Case 2 (Figure 5).

Pittsburgh compound B, used in amyloid scanning, is a radioactive tracer that binds to amyloid plaques in the brain. It is quantified in Centiloid units, with lower values indicating amyloid negativity. The presence of amyloid plaques can help make an AD diagnosis, although cerebrospinal fluid analysis or another measure of tau is re needed to clarify the diagnosis in some cases, as in Case 1 (Figure 4). In this person, the amyloid scan was positive, but the spinal fluid analysis was negative for AD. In addition, amyloid scanning helps determine treatment response and guide further management, as in Case 6 (Figure 9).

Tau PET scans, although not yet commercially available, hold promise for stage-specific disease quantification in AD.

## Cerebrospinal fluid and blood-based biomarkers

Biomarkers in the cerebrospinal fluid (CSF), including reduced levels of Aβ, increased total tau, and increased abnormally phosphorylated tau (p-tau) levels may be used in lieu of tau and amyloid PET imaging to make a biomarker-based diagnosis. Low CSF levels of Aβ in conjunction with high p-tau levels are consistent with a diagnosis of AD. Even in cognitively normal persons, 80% of those with abnormal CSF biomarkers progress to mild cognitive impairment in 6 years, while 90% of those with cognitive impairment develop dementia within a decade (Buchhave, 2012).

Increased CSF levels of neurogranin and neural filament light chain (NFL) protein are seen in several types of neurodegeneration. The CSF biomarker profile in AD differs from that of FTD, and allows for a different treatment approach, as up to 20% of patients with a clinical diagnosis of FTD are found to have AD by biomarkers (Padovani et al., 2013; Schöll et al., 2019; Mattsson-Carlgren et al., 2022). Using polypropylene tubes which do not bind and falsely lower Aβ levels is important in CSF analysis. Glass tubes may be used if polypropylene tubes are not available.

Standardization of cutoff values for CSF varies depending on the assays used. The commercial laboratory values for diagnosing AD in the United States are conservative, with AD diagnosed when values for Aβ42 are <700 ng/L, total tau >400 ng/L, and p-tau >54 ng/L, with a Aβ42/p-tau181 ratio of >0.8 (Blennow et al., 2015). In a Spanish study, the best cutoff values for diagnosing AD were Aβ42 of 750 ng/L, a total tau of 522 ng/L, and a p-tau181 of 70 ng/L (Puig-Pijoan et al., 2022). In a cohort of European memory disorder centers, high-cutoff values for diagnosing AD found Aβ42 ranging from 613 to 978 ng/L, t-tau ranging from 228 to 421 ng/L, and p-tau-181 ranging from 20 to 75 ng/L (Dumurgier et al., 2022).

While CSF access is widely available, methods of analyzing protein, and standardization of abnormal values are variable. Disadvantages of CSF biomarkers include the invasiveness of a lumbar puncture, and while quantification of the plaque and tangle load is achieved, brain areas affected by pathology are better visualized with neuroimaging. Neuroimaging, on the other hand, is extremely expensive because of the cost of the radioisotopes used and the need for two separate procedures, one for imaging amyloid and another for imaging tangles.

Blood-based biomarkers, including measurement of Aβ42 and tau, maybe the future of biomarker-based diagnosis of AD, given the ease of testing and lower costs involved (Leuzy et al., 2022). In Case 2 (Figure 5), blood-based Aβ42 results were used in conjunction with cerebrospinal fluid analysis to make a diagnosis. Blood levels of neurofilament light chain (NFL), glial fibrillary acid protein (GFAP), a marker of astroglial injury, and neurogranin, are increased in blood in neurodegeneration, although these are not specific to AD (Hampel et al., 2018; Hawksworth et al., 2022; Leuzy et al., 2022).

## Genetic testing

Commercial genetic testing panels for younger-age AD screen for mutations in the transmembrane PSEN 1 gene, PSEN 2 gene, and the APP gene. As sporadic cases with *de novo* mutations can occur, it is important to screen all younger-age AD cases for known mutations (Lanoiselée et al., 2017). There are currently over 300 mutations of the transmembrane PSEN1 gene and 38 mutations of the PSEN2 gene leading to AD, both with autosomal dominant inheritance, although mutations of PSEN2 exhibit variable penetrance (Plotkin and Cashman, 2020). The number of known mutations continues to grow, making genetic screens for younger-age AD useful when positive, but not when negative, as an as-yet unidentified mutation may be responsible.

APOE genotyping is helpful in both younger- and older-age AD. The presence of one copy of the ε4 allele increases the risk for older-age AD 3–4-fold when compared with ε3 carriers, with two copies increasing the risk 12–15-fold. This is clinically relevant for two reasons. For a cognitively asymptomatic person, one or two copies of the ε4allele would warrant closer, more biomarker-based monitoring over time for the development of the disease. For a symptomatic person who is a candidate for anti-amyloid monoclonal antibody therapy, APOE genotyping is essential, as patients with an ε4 allele are more likely to have more brain bleeding and edema necessitating modifications in dosage and titration (Salloway et al., 2022; Devi, 2023).

## Other tests

An electroencephalogram (EEG) may help aid in diagnosis, particularly when biomarkers are unavailable, and in following progression (Briel et al., 1999; Houmani et al., 2018). Early in the course of AD, there is an increase in theta and delta slow wave activity. As the illness progresses, there is an anterior drift of alpha activity from the occipital region as well as a reduction in amplitude and increasing slower rhythms (Briel et al., 1999; Houmani et al., 2018). In Case 1 (Figure 4), the EEG helped clarify diagnosis even in the absence of biomarkers, with no discernible EEG changes over time, which would be unusual for a patient with Alzheimer's disease, where there is generally progressively slower activity. Transcranial dopplers (TCD) may be of benefit in assessing vascular compromise in patients with dementia (Roher et al., 2011; Vinciguerra et al., 2019).

## Neuropathologic and neurocognitive subtypes of AD

Based on the pattern of tau pathology and regional areas of MRI atrophy, four subtypes of AD exist, including typical, limbic predominant, hippocampal sparing, and minimal atrophy AD (Murray et al., 2011; Ferreira et al., 2020). Typical AD, with tau pathology in both hippocampal and association cortices, is the most common with 55% of cases, while the other three each comprise approximately one-third of the remainder. There is a difference in age of onset, disease duration, and prognosis between the subtypes. The hippocampal-sparing subtype of AD is most often associated with younger age, earlier motor symptoms, and a more aggressive course, and is less associated with the ε4 allele. The limbic-predominant subtype is associated with a more indolent progression and the longest period to death (Ferreira et al., 2020). The limbic predominant subtype is more affected by TDP-43 pathology. Women are more likely to be affected by the limbic predominant subtype. Hippocampal-sparing AD patients decline at the rate of 5 points on the MMSE yearly, limbic predominant AD patients by 1 point annually and typical AD patients by 3 points annually (Murray et al., 2011).

Analysis of neurocognitive profiles reveals as many as eight neurocognitive clusters of Alzheimer's based on cognitive areas affected, with distinct demographics, symptoms, and progression (Scheltens et al., 2016). Memory-impaired clusters progressed more slowly and were associated with the ε4 genotype while the memory-spared clusters with visuospatial impairment had onset at a younger age and progressed more rapidly. Given the vast heterogeneity of AD, only a precise and meticulous mapping of findings to each individual patient can guide diagnosis, prognosis, and therapy (Devi and Scheltens, 2018).

## The informing visit (after workup is complete)

This is a key step in the care of a person with Alzheimer's disease or any other dementia. This visit provides information about the results of testing and how the diagnosis was arrived at. Information is provided about the relative certainty of diagnosis including the role and reliability of biomarkers. For example, in a person with evidence of cognitive impairment on standardized testing, with evidence of small vessel disease on neuroimaging, along with significant hippocampal atrophy and evidence of significant deposits of amyloid on imaging, the diagnosis of dementia would be attributable to several conditions, including cerebrovascular disease, hippocampal sclerosis, and AD, although a definitive diagnosis of AD would not be possible until tau load was assessed. This attention to tau is a departure from the current recommendation for treatment with monoclonal antibodies, which requires only a positive amyloid scan for implementation. But, as seen in Case 1 (Figure 4), a patient may present with profound and progressive cognitive impairment over time, with amyloid deposition, but without tau abnormalities. Based on current diagnostic guidelines for AD, requiring the presence of both plaques and tangles, this patient does not have AD and would not be appropriate for treatment with an anti-amyloid monoclonal antibody.

This is also the visit where the likely subtype of AD may be discussed, which has prognostic implications for patients and their families. Genetic risk for family members may be brought up then, and it is important to factor in time for such discussions. Patients and families inquire about the level of severity, or stage, of Alzheimer's disease. Early during AD, the impairment may stabilize for years. Given the heterogeneity of AD, as well as the co-pathologies involved, including primary brain and systemic and psychiatric co-pathology, it is generally difficult to prognosticate with any degree of accuracy.

The author has found, over 30 years of clinical experience, that it may be more helpful to stage the various cognitive areas, primarily, visuospatial, language, praxis, social, verbal memory, visual memory, and motor abilities separately, rather than assign an overarching stage. Patients progress along different trajectories in different cognitive arenas, making general staging neither accurate nor helpful. Monitoring response to treatment with neurocognitive data over at least a year to 2 years allows some measure of precision in prognosis.

It is also helpful to the patient, the family, and the physician to get a sense of overall life expectancy at some point early in the course of treatment. Life expectancy calculators are available online through life insurance companies and while they do not factor in neurodegenerative diseases, they reasonably estimate length of life, based on demographics and systemic comorbidities. This allows for planning for the individual patient, including financial and caregiving resources. General disease staging is best used at the advanced and terminal stages to help plan end-of-life care, including hospice and preferences regarding hospitalization, although these discussions should be started early in the course of treatment, so that patients can also participate.

## Treatment of Alzheimer's disease

Optimal treatment of AD may be conceptualized as treatment of five broad categories, primary treatment of Alzheimer's, treatment of co-morbid brain pathology, treatment of psychiatric co-morbidities, treatment of systemic co-morbidities, and socio-behavioral treatment.

### Primary interventions for treating Alzheimer's

Primary interventions for the treatment of Alzheimer's are those interventions approved for symptomatic use, treatment that modifies disease trajectory, and a third category of “off-label” interventions, which are not approved for treating AD, but may be of benefit. Over time, it has become clear that interventions traditionally viewed as symptomatic may have disease-modifying effects, including significantly prolonging time to nursing home placement. This is biologically plausible when one accepts that neuroplasticity, although attenuated, is still present in patients with AD, and changing activity in circuits by symptomatic treatment likely strengthens those neural circuits.

### Symptomatic medications

Cholinesterase inhibitors such as donepezil, galantamine, and rivastigmine may help slow cognitive decline and reduce mortality for as long as 6 years in patients with AD and other dementias (Xu et al., 2021; Zuin et al., 2022). Galantamine may be superior in slowing progression to severe dementia (Xu et al., 2021). It has been the author's practice to maintain the patient on a cholinesterase inhibitor for as long as feasible. Side effects occur even after several years of treatment, although they generally occur in the first few months of titration. They include nightmares and vivid dreams, cramps of the lower extremities, nausea, loose bowels, diarrhea, and even bowel incontinence, fatigue, weight loss, and persistent rhinorrhea. Rhinorrhea, not a widely known side effect, besets a significant number of patients on cholinesterase inhibitors who may then seek specialist consultations and procedures if not made aware of this side effect.

Side effects, present in 5–10% of patients, may be ameliorated by slower titrations over 3 months. Switching to another cholinesterase inhibitor may allay refractory side effects, as may switching from an oral to a transdermal route (Darreh-Shori and Jelic, 2010). Switching evening to morning dosage may lessen nightmares and quinine water or magnesium may help with leg cramps. Dose reduction is another option.

Memantine, an N-methyl-D-aspartate (NMDA) receptor antagonist, slows cognitive decline in both mild and moderate AD and may have neuroprotective properties (Lipton, 2007; Wu et al., 2009). It is a non-competitive NMDA receptor antagonist, inhibiting the intracellular influx of calcium and reducing neuronal excitotoxicity. While the guidelines for memantine in the United States suggest beginning the drug once a patient has reached the moderate stage of AD, the author begins memantine, given its neuroprotective properties, as early as the mild cognitive impairment phase. Memantine may have some antidepressant and stimulant effects. It is best taken once a day, given its very long half-life. It is generally well-tolerated.

### Disease-modifying medications

Disease-modifying drugs include those that specifically target Aβ42 and p-tau production and accumulation either through active or passive immunization, or other modulation of the immune system. Non-targeted immune modulation with intravenous immunoglobulin (IVIg), showed early promise, clearing Aβ plaques and reducing the inflammatory response, although a large trial did not find benefit in AD despite significant decreases in Aβ42 (Relkin et al., 2009, 2017).

Active immunization with vaccines that elicit neutralizing antibodies or passive immunization via monoclonal antibodies targeted to specific epitopes has become the preferred strategy. In an early trial of an active vaccination against Aβ42, aborted due to increased mortality, found that 4.6 years later, more patients who had been on drugs than on placebo lived at home (Vellas et al., 2009). Even so, by 15 years, all five patients with complete plaque clearance had progressed to severe dementia (Vellas et al., 2009). Passive immunity with targeted monoclonal antibodies (MAB) to Aβ42 ultimately yielded the two currently approved agents targeting Aβ42. Anti-p-tau MABs are in development.

## Reducing Aβ plaques: monoclonal antibodies to Aβ

Passive immunization with MABs to various Aβ epitopes all reduce brain amyloid deposits, many with downstream reduction in tau, and all with trends for potential clinical benefit over placebo. However, only two have shown significant clinical benefits over placebo in phase 3 trials. It is important to note that the outcome variables in all these trials have been historical and observational data, rather than objective measures, including the commonly used clinical dementia rating sum of boxes-18 (CDR-SB18) (Cummings et al., 2021; Mintun et al., 2021; van Dyck et al., 2022). The reduction in deterioration for drug vs. placebo was 0.4/18 points over 18 months with aducanumab and 0.45/18 points over 18 months with lecanemab on the CDR-SB18, for the two MABs currently approved for treating mild AD (Cummings et al., 2021) (Table 2). These differences are small, but the hope is that benefit accumulates over time.

**Table 2:** Selected anti-amyloid monoclonal antibodies.

**Drug**	**Aducanumab; approved**	**Lecanemab; approved**	**Donanemab; pending approval**
Binding	Fibrils>oligomers	Protofibrils 75–300 kDa	Fibrils, no oligomers
Inclusion	+Amyloid PET	+Amyloid PET	+Amyloid PET and +tau PET
MMSE	24–30	22–30	20–28
Number of patients	Phase 3; 3285, half to the drug, half on placebo; 3, 6, 10 mg doses	Phase 3; 1,800 pts, half to the drug, half on placebo.	Phase 2: 257 pts; 130+/group; 93/group completed trial
Length	18 months	18 months	18 months
Dosing	10 mg/kg q m, 1 mg to10 mg titrated over 8 m	10 mg/kg biweekly, no titration	700 mg q m for 3m, then 1,400 mg q m.
Plaque Reduction	44 CL↓ in 26 wks, 50% plaque-, 57 CL↓ in 18 m	70% plaque-, 59 CL↓ in 18 m	40% plaque-, 68 CL↓ in 6 m; 70% plaque-, 85 CL↓ in 18 m
Clinical outcome	0.4 vs. placebo on 18-pt CDR-SB;10 mg dose	0.45 vs. placebo on 18-pt CDR-SB	3 pts vs. placebo, iADRS; no change on18-pt CDR-SB
ARIA H	34% 10 mg dose	17%	31%
ARIA E	35% 10 mg dose	13%	27%
ARIA E/ H	41% 10 mg dose	21%	40%

ARIA, Amyloid Related Imaging Abnormalities; ARIA H; Amyloid Related Imaging Abnormalities hemorrhage; ARIA E; Amyloid Related Imaging Abnormalities edema; MMSE, Mini-mental state examination; PET, Positron Emission Tomography; CL, Centiloid units to measure plaques; CDR-SB, Clinical dementia rating scale- sum of boxes 18-point scale of cognitive ability, higher score more impairment; iADRS, integrated Alzheimer's Disease Rating Scale is a 144-point scale of cognitive ability, lower score more impairment.

Aducanumab targets oligomers and amyloid plaques. Lecanemab preferentially targets soluble protofibrils (large oligomers) over plaques (Gandy and Ehrlich, 2023). Both reduce downstream tau. Of note, both aducanumab and lecanemab cause amyloid-related imaging abnormalities (ARIA), with brain edema and microhemorrhages related to plaque dissolution, and both are parenterally administered. Some level of cerebral amyloid angiopathy, with amyloid deposits in parenchymal and leptomeningeal arteries and capillaries is seen in 85–95% of AD patients (Deture and Dickson, 2019). Anti-amyloid immunization strategies may affect the cerebral amyloid angiopathy-related risk of ARIA (Deture and Dickson, 2019). A slower dose titration, further modified by the presence of the ε4 allele, appears to significantly lessen aducanumab-related ARIA, possibly related to reduced speed of clearance (Devi, 2023).

Donanemab, likely to be approved shortly, targets only plaques (Mintun et al., 2021). Lecanemab is the only drug thus far evaluated in patients on anticoagulation, with a 2.4% macro hemorrhage rate (van Dyck et al., 2022). A single MAB targeting a single epitope may not be able to reduce pathology optimally, but combination therapy with multiple MABs has not yet been described.

**Figure 7:**
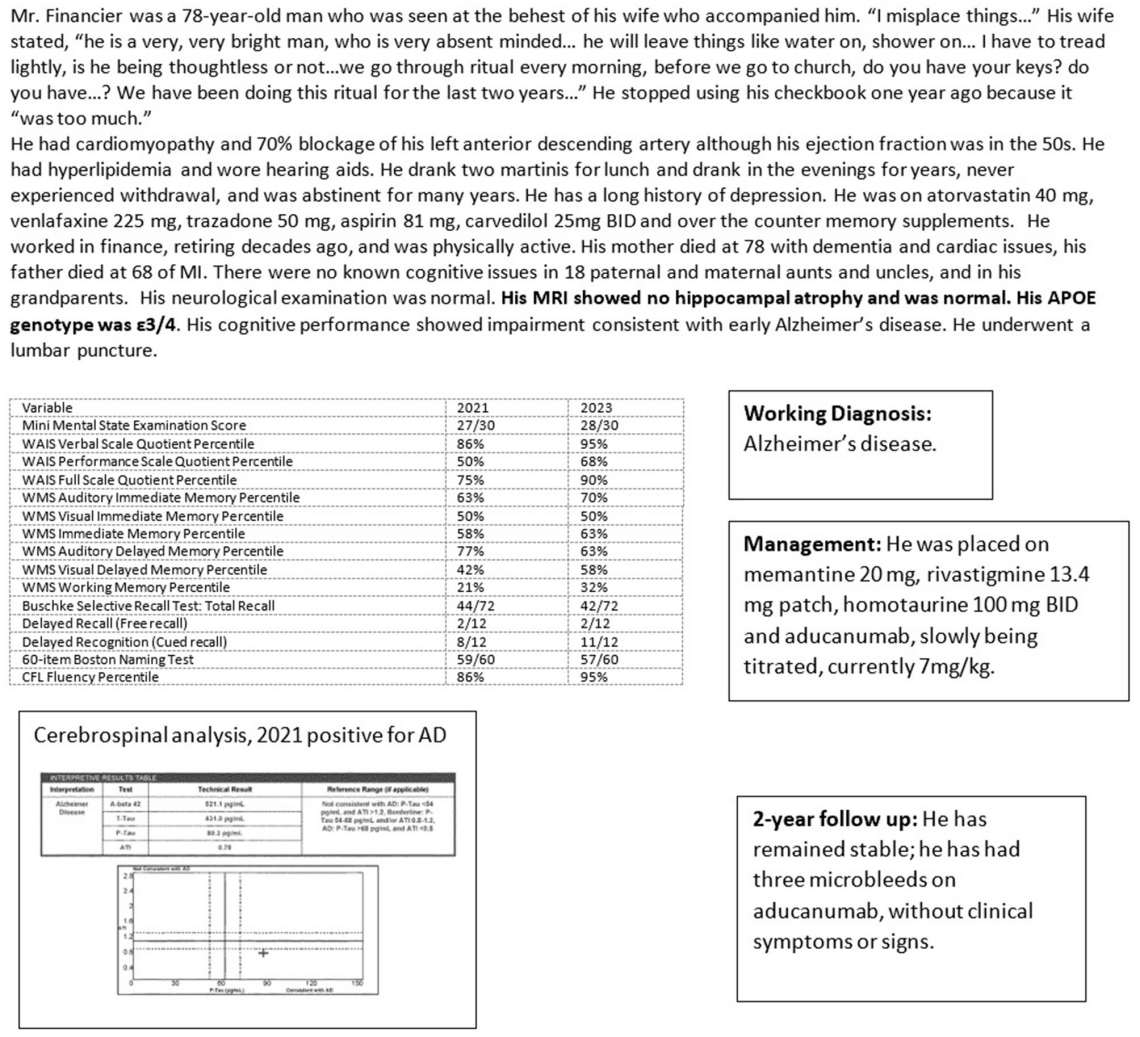
Case 4—Alzheimer's treatment: oral medication and intravenous aducanumab.

Case 4 (Figure 7) illustrates the treatment of a patient with Alzheimer's confirmed by biomarkers, with oral medication as well as intravenous aducanumab on a slow titration schedule. He developed three asymptomatic microhemorrhages, discovered on periodic neuroimaging mandated by the drug protocol. He has been stable on this disease-modifying regimen.

## Reducing tau

Tau immunotherapy using humanized anti-tau MABs has shown less promise. Semorinemab, one of several anti-tau MABs to have entered clinical trials, failed to stem tau deposition despite extremely high doses (Teng et al., 2022). The therapeutic rationale of this approach is that the MAB intercepts extracellular tau seeds, inhibiting the spread of tau pathology. However, as tau is primarily intracellular, MABs that do not reach cytosolic tau may not be effective (Sandusky-Beltran and Sigurdsson, 2020). Additionally, pathogenic tau species differ between patients, possibly requiring patient-specific anti-tau MABs for effective anti-tau antibody treatment (Dujardin et al., 2020). Nevertheless, given the extent that tau pathology tracks disease onset and progression, the development of effective anti-tau MABs for treating AD remains an important goal.

## Off-label interventions with biological plausibility for use in AD

### Estrogen and other off-label medications

The use of estrogen in women as an adjunct in treating Alzheimer's disease has a strong neurobiological rationale but has had variable results, with only some studies finding benefits, particularly with estradiol, a bioequivalent estrogen (Wharton et al., 2011; Song et al., 2020; Saleh et al., 2023). One factor responsible for the variability in response may be the presence of the ε4 allele (Taxier et al., 2022; Saleh et al., 2023). Apolipoprotein E is the main cholesterol transporter in the brain and women with an ε4 allele may benefit less from estrogen therapy due to a possible inefficiency in transporting estrogen into nerve cells (Taxier et al., 2022). While the consensus recommendation is not to use estrogen therapy for treating Alzheimer's disease, the author has used estrogen as adjunctive therapy in some women, using the n-of-1 approach. Earlier age at menopause and later initiation of hormone therapy was found to increase vulnerability to tau in the presence of elevated Aβ in postmenopausal women, suggesting a protective role for estrogen (Coughlan et al., 2023).

Several common medications are being investigated for potential benefits as adjunctive treatments for Alzheimer's disease. They include antiviral agents, agents reducing insulin resistance, and immunomodulators. Interesting associations have been noted between AD pathogenesis and bacterial infections such as *Porphyromonas gingivalis*, the cause of periodontal disease, viral infections such as herpes simplex virus type 1, and commensal gut bacteria (Hardy et al., 2023). The wide variety of agents being investigated as possible treatments in AD range from amlodipine to metformin to valacyclovir and sirolimus (Cummings et al., 2022).

## Off-label neuromodulation: transcranial magnetic stimulation (TMS) and transcranial direct current stimulation (TDCS)

Neuromodulation, using transcranial magnetic stimulation (TMS) and transcranial direct current stimulation (TDCS), may be a promising adjunctive treatment in patients with AD (Gonsalvez et al., 2017; Holczer et al., 2020). TMS induces a magnetic field to either excite or inhibit a cortical area of ‘1 cm^2^, modulating long-term cortical excitability by inducing changes in synaptic circuits. With TDCS, a fixed 1–2-mA current modulates brain activity. While TDCS is portable, inexpensive, and may be administered at home, focusing on a specific brain region is more feasible with TMS.

Several studies have found that neuromodulation improved cognition in patients with Alzheimer's, with additional benefits from medication or cognitive training, while others have found no beneficial effect (Holczer et al., 2020; Wang et al., 2020). A multi-national European task force found only level 3 evidence for TMS in AD, and it is not currently an approved treatment for AD (Lefaucheur et al., 2020).

The outcome of neuromodulation in treating AD is influenced by various patient and stimulation-specific parameters (Gonsalvez et al., 2017). An additional major issue with interpreting data in neuromodulation trials is the problem of the sham control, as patients can nearly always identify active treatment (Burke et al., 2019). In the author's experience, neuronavigation-guided TMS therapy may be of benefit, particularly for maintaining non-memory functions such as language, as seen in cases 1, 5, and 6 (Devi et al., 2014; Tumasian and Devi, 2021).

**Figure 8:**
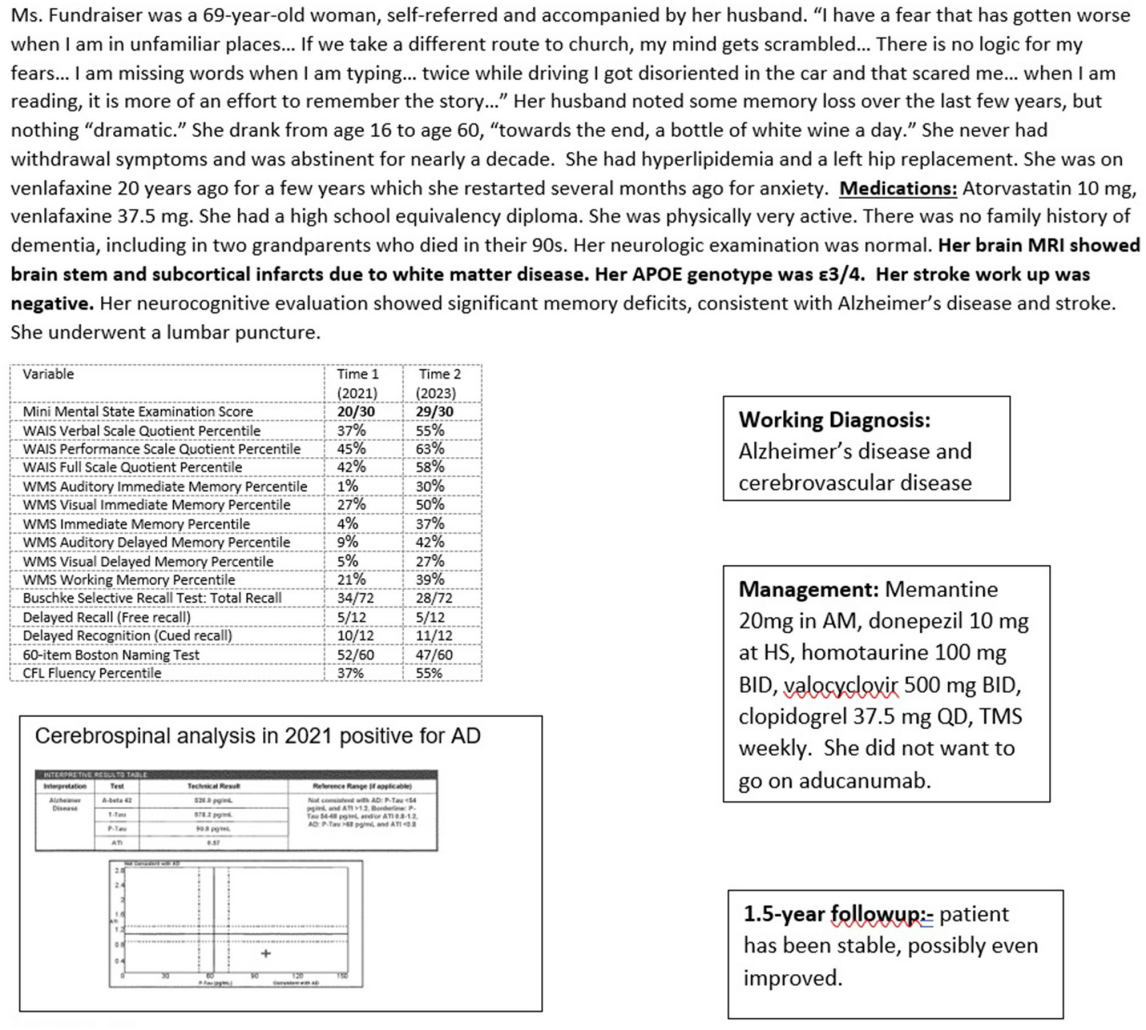
Case 5—Alzheimer's treatment: oral medication and transcranial magnetic stimulation.

Case 5 (Figure 8) is a patient with AD and stroke treated with oral medications and TMS. She improved over 15 months, with her MMSE going from 20/30 to 29/30. While she had a history of chronic, low-level depression, a potential target for TMS, the depression did not appear to have affected her neurocognitive performance.

### Treatment of co-morbid brain pathology

Comorbid brain pathologies in AD that can currently be addressed include cerebrovascular pathology, Parkinson's disease, and normal pressure hydrocephalus. Aggressive management of risk factors for cerebrovascular disease including treating hypertension, and hyperlipidemia reduces the possibility of future stroke and concomitant cognitive decline. A stroke workup is recommended in patients with significant cerebrovascular disease. Interventional treatment options for cardioembolic sources of stroke include cardiac ablation and the use of indwelling implants to close the left atrial appendage and prevent blood stasis, clot formation, and the risk of embolic stroke. Antiplatelet agents and anticoagulants are alternatives, although the increased risk of intraparenchymal hemorrhage with these drugs may preclude the concomitant use of anti-amyloid MABs.

In PD, using a monoamine oxidase inhibitor to delay symptom onset or levodopa to help with symptoms is beneficial (Fabbrini et al., 2012). In patients with NPH, the use of shunting helps with management, particularly gait imbalance, but those patients who benefit from shunting may also be more likely to have concomitant AD (Müller-Schmitz et al., 2020).

### Treatment of psychiatric co-morbidities

Anxiety and depression in patients with AD should be treated aggressively as the benefits extend beyond alleviating psychiatric symptoms. Patients often report better cognition, improved social interaction, and increased compliance with activities of daily living, which can alleviate caregiver burden. Selective serotonin reuptake inhibitors such as escitalopram are well-tolerated and effective in treating these symptoms (Knopman et al., 2021).

When aphasia and ideomotor apraxia become prominent, the patient may become anxious and agitated, perhaps not understanding what a “shower” means, and fighting when directed into it. While “agitation” is a commonly used term in these instances, it is non-specific, and it is more helpful to describe the circumstances surrounding such a response. Does the patient “get agitated” when made to change clothing or to made exercise? This may allow the physician to better understand the underlying cause and more effectively address it.

Agitation in AD is nearly always the behavioral expression of underlying anxiety and may resolve with antidepressant treatment. Psychosis, including paranoid ideations often involving caregivers, needs to be aggressively addressed. Disinhibition may also be an issue. Atypical antipsychotic agents such as olanzapine and quetiapine may be helpful, but it is essential to discuss the relevant “black box” warnings with the patient and family. Valproic acid, found by the author to be beneficial in some patients refractory to other medications, was found ineffective in a recent review (Baillon et al., 2018). Pimavanserin, an atypical antipsychotic agent approved for treating Parkinson's psychosis, is a choice in patients prone to developing extrapyramidal symptoms, such as those with Lewy body dementia. Very expensive, it may not be a viable option for many patients (Meltzer et al., 2010).

Sleep disturbances in AD patients may be addressed by low doses of antidepressants such as trazodone, mirtazapine, or doxepin, or melatonin-enhancing drugs such as ramelteon. Suvorexant binds orexin, a neuropeptide that promotes wakefulness and may be of benefit for insomnia in patients with Alzheimer's (Herring et al., 2020). Interestingly, suvorexant also reduces the level of p-tau and Aβ (Lucey et al., 2023). Purely anticholinergic medications such as diphenhydramine and all benzodiazepines should be avoided for treating agitation and insomnia. They may lead to tolerance, paradoxical effects, and detrimental effects on cognition. Sleep apnea, if a cause of insomnia, should be addressed. If weight contributes to sleep apnea, this should be preferentially addressed as the consistent use of a mask apparatus is challenging in patients with AD.

Overall, the treatment of psychiatric symptoms in AD patients is crucial not only for improving quality of life, but also for reducing caregiver burden. A personalized approach should be taken, considering the individual's medical history, medication use, and potential side effects. It is also important to regularly monitor and reassess treatment effectiveness to make any necessary adjustments to medications and dosages.

### Treatment of systemic comorbidities

Various other strategies addressing systemic comorbidities help treat patients with AD. Intensive blood pressure control, with a target systolic blood pressure < 120 mm Hg, is effective in reducing the risk of cognitive impairment compared to standard blood pressure control (Williamson et al., 2019).

A long-term, randomized Finnish trial found that a multidomain lifestyle-based intervention, including a program of healthy balanced nutrition, physical exercise, cognitive training, social activities, and vascular and metabolic risk management, was effective in reducing the risk of cognitive impairment. This was true in individuals with genetic risk factors for AD (Kivipelto et al., 2018). Such an approach is also beneficial in slowing decline in patients with AD.

Cholesterol-lowering agents such as statins reduce the risk of AD, possibly through a direct effect on APP processing, in addition to reducing the risk of cerebrovascular disease (Langness et al., 2021). Hearing and visual loss should be addressed and treated aggressively as it increases social isolation in patients. However, the proper use of hearing aids may be difficult for some patients. Given the increased risk for delirium and other morbidity, hospitalizations are best avoided or shortened in patients with AD with as much as possible. The workup and care are better rendered in an outpatient setting. Urinary tract infections, rampant in women with AD, may be prevented with ongoing low-dose antibiotics and/or vaginal estrogen. Baseline bone density evaluations are recommended in both men and women, and aggressive treatment of osteopenia and osteoporosis prevents the risk of fall-related fractures.

### Socio-behavioral treatment

A diagnosis of Alzheimer's disease does not necessarily mean that an individual must stop working, even in cognitively demanding positions as physicians or judges (Devi, 2018a). Recommendations regarding the ongoing ability to work should be based on the specifics of the individual circumstance, including the particulars of the neurocognitive performance, rather than on the diagnosis. Patients with other varied diagnoses such as Parkinson's disease, multiple sclerosis, depression, or stroke may be cognitively far more compromised than patients in the earlier stages of Alzheimer's disease. The ability to continue working helps patients maintain cognitive resilience and confidence, and contribute to society.

Multimodal interventions involving diet and cognitive and physical engagement help improve cognition (Isaacson et al., 2019). Anti-inflammatory, pro-cardiac health diets with low levels of carbohydrates and fats may be helpful in both AD prevention as well as reducing ongoing inflammation, a known driver of pathology in AD (Charisis et al., 2021). Physical therapy or working with a trainer can be helpful for patients, but it may not work for everyone, particularly those with abulia and lack of initiative. Regular aerobic exercise has been consistently shown to help cognition and may slow disease progression (Devanand et al., 2023). Exercise releases myokines, increasing the expression of brain-derived neurotrophic factor, which promotes hippocampal neurogenesis and increases synaptic plasticity (Benarroch, 2022).

Cognitive exercises are beneficial in maintaining functioning and strengthening cognitive reserve (Belleville et al., 2023). Some patients find these exercises to be anxiety-provoking and depressing. Similarly, support groups such as those provided by the Alzheimer's Association may be helpful for some patients, but for others, interacting with more impaired group members may increase unease and fear over their own future. Therefore, while theoretically beneficial for all patients, these interventions may not be practical for some.

It is essential to provide ongoing education to both patients and caregivers about what to expect in terms of prognosis and care over the ensuing years. However, a directive such as “go home and put your affairs in order” is needlessly alarming, depressing, and unhelpful. Instead, it is essential to provide information in a compassionate and supportive manner to help patients and caregivers plan and prepare for future.

## A biologically complementary, multimodal approach to treating Alzheimer's disease

**Figure 9:**
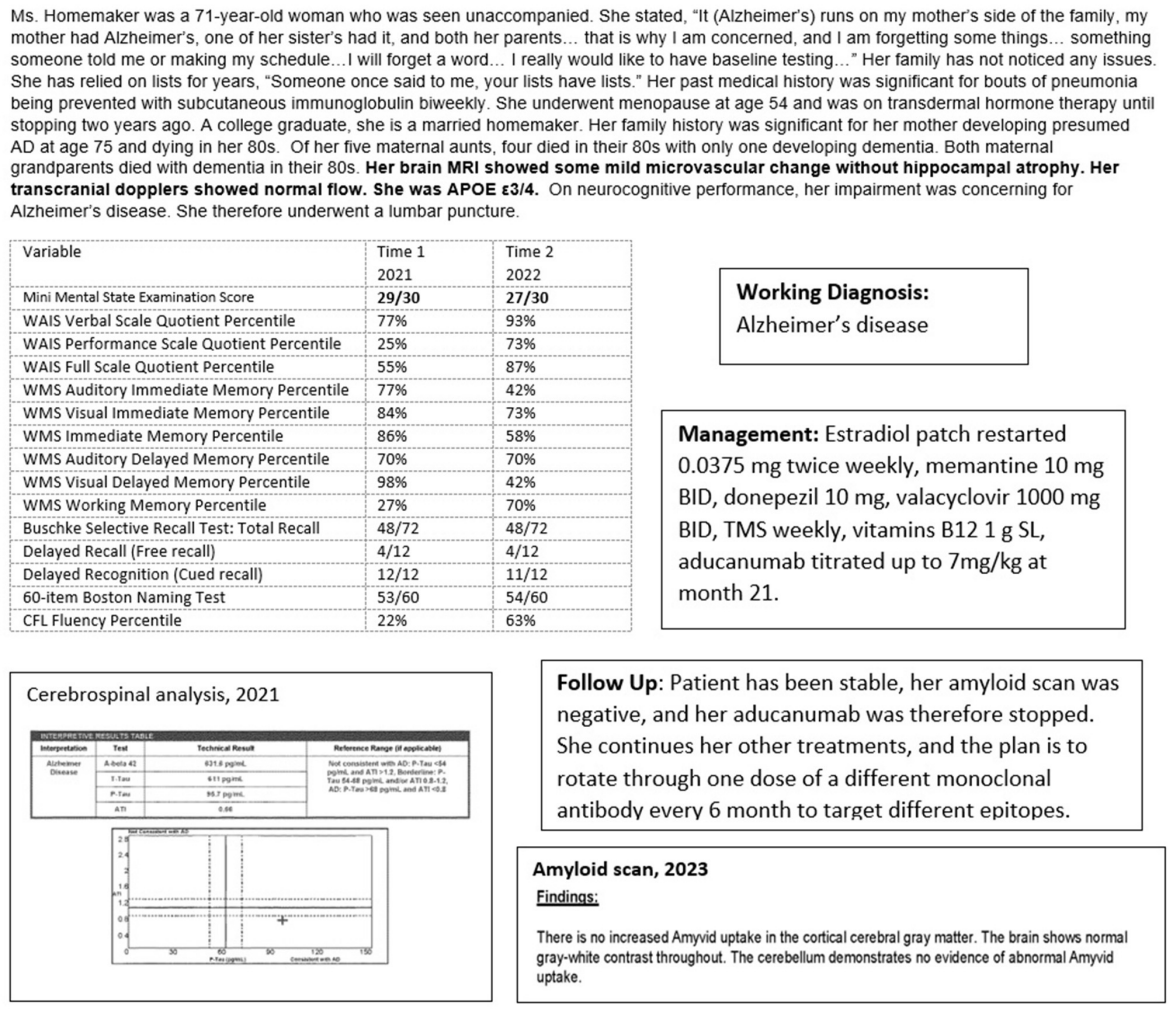
Case 6—Alzheimer's treatment: oral medication, aducanumab, and transcranial magnetic stimulation.

Case 6 (Figure 9) illustrates the complementary multimodal approach to the treatment of AD. The patient, a 71-year-old woman, had no cognitive complaints but wanted a baseline evaluation given a strong maternal family history of older-age dementia, presumed to be AD (no biomarker or autopsy confirmation). Her genotype was APOE3/4 and she had significant impairment on her neurocognitive evaluation, with a brain MRI showing mild vascular changes and normal transcranial dopplers and electroencephalogram.

However, her cognitive evaluation showed significant deficits in memory and language, despite scoring 29/30 on the MMSE. She scored 4/12 on a delayed recall list-learning task and verbal fluency at the 22nd percentile, despite excellent verbal skills. This was concerning for early Alzheimer's disease. She underwent a lumbar puncture which was consistent with a diagnosis of Alzheimer's disease with a low Aβ42 of 632 ng/L, consistent with Aβ42 deposition in the brain, and a high p-tau level of 96 ng/L.

She was informed of her diagnosis of early Alzheimer's disease, and an aggressive treatment regimen to prevent progression was recommended. She embarked on a course of approved oral medications for treating AD, off-label oral medication for treatment of her AD (including estrogen replacement and valacyclovir), off-label weekly neuronavigation guided transcranial magnetic stimulation of her left dorsolateral prefrontal cortex, Broca's, Wernicke's, and biparietal cortices, and a slowly titrated monthly infusion of aducanumab. Repeat cognitive testing at 14 months showed overall stability with improvement in language and visuospatial functions. She continued to function independently at home. Her weight was optimal with good dietary habits. She was physically and socially active. While she would have benefited from cognitive exercises, these were discontinued as she became very anxious during the sessions.

Amyloid scanning was performed 22 months into treatment with aducanumab, primarily for consideration of a switch to the recently approved lecanemab. She has become plaque negative. Aducanumab was therefore stopped and she continues her other treatments. The plan will be infusions every 4 to 6 months of available monoclonal antibody therapies on a rotating basis, with ongoing objective monitoring of her cognitive status. While the therapeutic value of rotating monoclonal antibodies to target different Aβ oligomer epitopes is speculative, the goal is to ultimately allay oligomer-driven neurotoxicity.

## Conclusion and future directions

Precision medicine is particularly relevant for treating Alzheimer's disease, given not only the tremendous variability in clinicopathology but also the inherent inter-individual variability of the human brain. Given the vast heterogeneity of AD, its coexistence with other primary brain co-pathologies, psychiatric and systemic co-morbidities, it is unlikely that an effective therapeutic approach for one type of Alzheimer's disease, or one subtype, may be beneficial for another. It is essential to tailor treatment and approach prognosis on a detailed, case-by-case basis.

## Author contributions

The author confirms being the sole contributor of this work and has approved it for publication.
